# An Assessment of Public Knowledge and Potential Health Impacts of Global Warming in Ghana

**DOI:** 10.1155/2020/7804692

**Published:** 2020-12-09

**Authors:** Stephen T. Odonkor, Anthony M. Sallar

**Affiliations:** ^1^School of Public Service and Governance, Ghana Institute of Management and Public Administration Accra, Ghana; ^2^School of Liberal & Social Sciences, Ghana Institute of Management and Public Administration Accra, Ghana

## Abstract

Global warming is a serious threat to human existence. The relatively higher level of global warming in recent times poses higher health risks to humans, both directly and indirectly. The aim of the study was to investigate public knowledge of global warming and its effects on human health. A nationally representative survey of Ghanaian adults (*N* = 1130) was conducted from November 1, 2018 to February 28, 2019. Results show that 84.4% of the respondents understood the meaning of global warming. Respondents' perceived causes of global warming include natural processes, deforestation, act of the gods, burning of fossil fuel, and carbon dioxide (CO_2)_ emission from vehicles and industries. The majority of the respondents (83.4%) indicated that global warming has an impact on human health, while 8.5% indicated that it does not. Majority (78.6%) of the respondents are willing to support efforts to reduce the intensity of global warming. Television (19.1%) and social media (18.6%) were the leading preferred methods for receipt of global warming information. These findings provide useful insights for policy directions. The Government of Ghana and other stakeholders in health should develop a communication strategy to increase and sustain publicity and education of the citizenry on global warming.

## 1. Introduction

Global warming is undoubtedly a major problem with worldwide attention and focus. Its occurrence is as a result of the elevation in average global temperatures facilitated by the greenhouse effect [1–3]. According to Edenhofer [4], the earth has become warmer in the past three decades as compared to any decade before 1850. Unlike years before the 20^th^ century where global warming was significantly under control, managing this phenomenon has become an extremely difficult task to carry out in this 21^st^ century as a result of the rise in human-orchestrated industrial and power house emissions [5–7]. The relatively higher level of global warming in recent times poses higher health risks to humans, both directly and indirectly [8]. Global warming is linked to heat stress which can cause kidney stones, heat strokes, heat cramps, heat syncope, and heat exhaustion in humans [6, 9–12]. Also, global warming is associated with respiratory diseases such as asthma [13, 14]. Additionally, it can result in drought, crop failure, and an increase in vector and water—borne diseases which indirectly affect the health of humans ([3, 6])—thus, increasing chances of high mortality among humans.

Due to the worldwide magnitude and alarming nature of global warming in recent years, knowledge of its public health ramifications is rapidly increasing; though research into this area is new [15–17]. In some countries, official reports and campaigns have been rolled out specifically to increase public awareness on the health risks associated with global warming. The United States, for instance, through their national climate programmes, has churned out key points to educate her people on climate change and health [18]. In Sub-Saharan Africa, Nigeria and Ghana are among several other nations that have both set up climate change adaptation policies to protect their environment and citizens [19, 20].

In public health practices, it is critical for people to be knowledgeable about dangers and risks to their health and wellbeing [21, 22]. Thus, people need to understand the health risks associated with global warming, so they can embark on relevant procedures to protect themselves and also actively participate in national mitigation agenda or activities. This will go a long way to sustain their health and physical wellbeing. It is not accurate or substantive enough to assume the public are well informed about global warming, just because of its policies and worldwide publicity. Sufficient evidence exists to attest to this. For example, Miabach et al. [16] found that although there is substantial general awareness among many fragments of the US population concerning global warming, only few Americans understood the various harms it causes. Similarly, adolescents in a study conducted in Indonesia demonstrated low knowledge concerning climate change and its health consequences among humans [14]. Hence, it is prudent to exclusively investigate people's knowledge concerning the global warming enigma to get a vivid representation of their understanding on its health ramifications.

Generally, impact studies relating to global warming and climate change is widely limited in developing nations like Ghana as compared to the developed world. Yet, Africa is known to be the most vulnerable region to climate change ([23–25]). Although climate change is not a problem of Africa's making, yet Africa stand to be particularly hard hit because of their geography, their agricultural dependence, and because of difficulties that adaptation will face. It is projected that by 2020, 85-250 million Africans will experience water stress as a result of climate change and crop yields from rain-fed agriculture may decrease up to 50%. This will be devastating because 30-40% of Africa's GDP and about three-quarters of its population relies on agricultural production as primary income sources [26, 27].

In Ghana, climate change is causing considerable variations in temperature and rainfall patterns. Rainfall regime has been shifting towards a longer dry season [28, 29]. Furthermore, the temperature has increased by 1°C across the country, representing an average increase of 0.21°C per decade [30]. Ghana is likely to experience greater rainfall variability and higher temperatures in the future. An increase in temperature averaging 0.25°C is expected from 2010 to 2020, while rain fall is projected to decrease in most of agroecological zones. Such changes will shorten the growing season with implications for the agricultural and fisheries sectors [31].

Global warming is estimated to negatively affect human health particularly in developing countries like Ghana by aggravating already existing health problems. This is further worsened by a weak healthcare system and poverty [18, 32]. However, baseline studies to investigate people's knowledge on health threats of global warming seem to be elusive in Ghana, thus, leaving a wide gap in global warming and climate change impact studies in Ghana. Yet, this remains critical to stakeholders, with regard to the environmental impact on the health of the citizenry.

The objective of the present study is to investigate public knowledge of global warming and its effects on human health. This is relevant in several ways. It will help evaluate people's understanding on global warming and its associated health implications which will aid in informing policies towards increasing public awareness and adaptation strategies for the global warming phenomenon. Additionally, it could aid in determining the general conception people have towards climate change health impacts, which is critical towards its mitigation [33, 34].

## 2. Materials and Methods

### 2.1. Research Design

The study employed a descriptive, cross-sectional design with self-administered questionnaires to assess the level of knowledge. The study sought to investigate public knowledge of the effects of global warming on human health in Ghana. The study was conducted from November 1, 2018 to February 28, 2019. Questionnaires were self-administered and took an average of 28 minutes to complete. The average margin of error (95% confidence interval) for the survey is “3 percentage points.

### 2.2. Survey Subjects and Technique

Data were obtained from a nationally representative survey of Ghanaian adults (*N* = 1130). The study utilized a stratified sampling technique. The country was demarcated into 3 zones: southern belt, middle belt, and northern belt. Therefore, in selecting the respondents for the survey, a sampling proportionate to size was utilized to determine the number of respondents to be interviewed form each zone. All adults 18 years and above present in the demarcated zone were considered for the study.

### 2.3. Survey Content

The study employed a standardized structured questionnaire intended to achieve the goals of the research for data collection. The constructs in the questionnaires were informed by literature with respect to global warming and its effect on human health. After each day's interviews, field inspection of questionnaire data was done. This allowed for immediate verification and correction of errors that were identified. The final survey instrument comprised 57 questions in eight thematic areas: sociodemographics (6 items), knowledge and understanding of global warming (9 items), concern about global and local environmental issues (8 items), perceived causes of global warming (6 items), effect of global warming on human health (8 items), causes of global warming (5 items), groups vulnerable to health impact of global warming (10 items), and preferred methods to receive information on global warming (5 items).

Six experts in social sciences measurement and evaluation determined the face validity of the instrument. The average overall face validity was equal to 95%. The study used Cronbach's alpha test for reliability testing, which yield a reliability coefficient of 0.8. Cronbach's alpha test assesses the internal consistency of a set of scales or items to ensure that they are all consistent in measuring the same attributes under study.

### 2.4. Ethical Consideration

Both verbal and written concerns were sought from the respondents before data was obtained. Adequate information was provided to the respondents with regard to the aims of the study. It was made clear to the respondents that their participation was voluntary and they were at liberty not to participate. They also were assured of confidentiality. All respondents' personal identifiers were deleted from summarized data, ensuring confidentiality. Ethical clearance was obtained from the Ethics Review Committee (ERC) of GIMPA School of Public Service and Governance.

### 2.5. Statistical Analysis

Data obtained from the questionnaires were coded and analysed with SPSS version 23. Discrete variables like gender and educational status were described using frequencies and percentages. Bivariate relationships were analysed using Chi-squared (*χ*^2^) tests. All statistical tests employed in this study were two tailed and were considered to be significant when *α* = 0.05 or less.

## 3. Results

Table 1 shows the demographic characteristics of the respondents. There were a total of 1130 respondents in this survey. There were more females (52.2%) than males (47.8%) in the study. In terms of age group, almost half (43.0%) of the respondents were between 20 and 30 age group, while age group 50 years and above had the least (3.7%) number of respondents. Most of the respondents (28.9%) had undergraduate qualification, 22.9% had primary education, 21.2% had no formal education, and 18.3% had secondary education. Majority of the respondents (59.3%) are from urban settlements, while 40.7% are from rural settlements. Almost half of the respondents (47.6%) were from the Middle belt, 35.4% were from the Coastal belt, and very few (17.0%) were from the Northern Belt of the country. However, in terms of their social status, more than half (52.9%) of the respondents were in the middle class, 37.7% were in the upper class and 9.4% were in the lower class.

Table 2 shows the respondents' knowledge and understanding of global warming. Almost all the respondents (91.3%) indicated that they have heard of global warming. However, 8.5% and 0.2% of the respondents had never heard and did not know of global warming, respectively. However, the number of respondents who said they have heard of global warming decreased to 84.4% when asked if they understood the meaning of global warming. Also, 12.2% and 3.4% responded that they do not understand or do not know the meaning of global warming.

Respondents' concern about environmental issues is presented in Table 3. Almost half of the respondents (49.2%) said they were very concerned about global environmental issues, 43.2% indicated they were concerned, 6.4% were not concerned, and 1.2% said they were not at all concerned about global environmental issues. However, more than half (55.4%) were very concerned about local environmental issues, while 39.5%, 3.9%, and 1.2% were concerned, not concerned, and not at all concerned, respectively, about local environmental issues.

**Figure 1:**
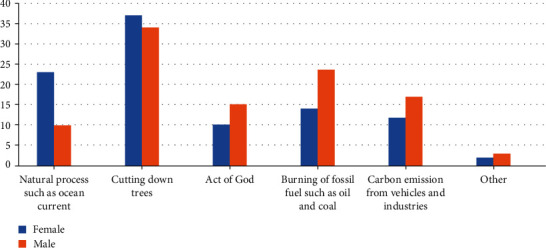
Respondents' perceived cause of global warming.

Figure 1 shows respondents' perceived causes of global warming. In response to the causes of global warming, more than one-third (37.9% and 34.07%) of female and male respondents, respectively, indicated that cutting down of trees was the main cause of global warming. Furthermore, 23.05% and 10.0% of female and male respondents, respectively, said natural process such as ocean current was the cause of global warming. Very few respondents (11.8% of females and 17.04% of males) said that carbon emissions from vehicles and industries were the causes of global warming. Interestingly, 10.17% and 15.19% of female and male respondents, respectively, said act of the God were the cause of global warming.

Table 4 shows the respondents' perceived effects of global warming on human health. Global warming may have a significant impact on human health. Majority of the respondents (83.4%) indicated that global warming has an impact on human health while 8.5% indicated that global warming does not have an impact on human health. However, 49.2% of the respondents indicated that global warming is very bad to human health, 34.7% indicated it was bad, 2.8% said it was good, while 2.1% and 11.2% said it was bad and do not know, respectively. It is worrying to note that, although majority of the respondents know global warming has an impact on human health, they are unaware of the degree of the impact to their health.

Table 5 shows the respondents' perceived effects of global warming. More than half (50.3%) of the respondents strongly agreed that heat stroke from extreme hot temperatures are caused by global warming, while 1.4% strongly disagreed. Moreover, 40% of the respondents strongly agreed that malnutrition/food reduction/hunger have been caused by global warming. Meanwhile, 4.6% strongly disagreed that it has been caused by global warming. In relation to whether the incidence of vectorborne illness such as malaria and cholera are affected by global warming, 38.6% agreed while 8.0% strongly disagreed. Meanwhile, 14.5% and 11.5% strongly agreed and strongly disagreed, respectively, that heart diseases are caused by global warming. In terms of whether contaminated water was caused by global warming, 40.5% and 3.0% strongly agreed and strongly disagreed, respectively. In terms of whether drought/water shortage/fires are caused by global warming, more than half of the respondents (51.2%) and 2.3% strongly agreed and strongly disagreed. However, 29.0% and 23.9% of the total respondents strongly agreed and strongly disagreed, respectively, that lung disease/asthma/respiratory problems are caused by global warming. More than half of the respondents (53.3%) and 1.2% strongly agreed and strongly disagreed that flooding and downpours are caused by global warming. In terms of whether mental health problems such as stress was caused by global warming, 14.0% strongly agreed while 31.5% strongly disagreed. However, in terms of whether pollution//air pollution/air quality have been caused by global warming, more than half (65.3%) of the respondents strongly agreed while 5.8% strongly disagreed.

Table 6 shows respondents' perception of groups vulnerable to health impact of global warming. Respondents were asked which group of people they think are vulnerable to the effects of global warming. Of the total number of respondents, 12.2% indicated people living with light sensitive skin, and 12.1% indicated the sick, disabled, obese and low immunity, and everyone. However, 11.7% indicated that infants were most vulnerable, while 5.5% of the respondents indicated they were not sure.

Table 7 shows the respondents' belief of what will become more or less common in their communities in the next 5-10 years if nothing is done about global warming. Although global warming has caused some environmental issues such as flooding, air pollution, and spread of waterborne diseases, respondents were asked of their view of what will happen if no action is taken in the future. Almost all the respondents (91.7%) indicated that air pollution would be much more common while 1.4% indicated air pollution would be a little less common. More than half (56.6%) of the respondents indicated asthma and other lung diseases would be much more common, while 3.7% indicated it would be a little less common. However, most of the respondents 71.3% and 3.7% stated that heat stroke from very hot waves would be much more common or a little less common, respectively.

Table 8 shows respondents' support for funding and their willingness to help support efforts to reduce global warming. Majority of the respondents (78.6%) indicated that due to the effect of global warming on weather-related conditions and their health, they are willing to support to reduce global warming intensity. However, 32.3% indicated they would not support any project to reduce global warming intensity. More than half of the respondents (58.4%) stated that increase funding to the region and district and other health agencies would help to protect against the health effect of global warming while 9.4% do not agree to this.

**Figure 2:**
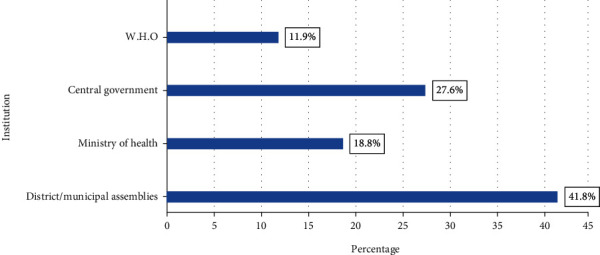
Which institution should be doing more to protect the public against the health effect of global warming?

Figure 2 shows the respondents' opinion of the institution that should be doing more to protect the public against the health effect of global warming. In relation to which institution is more responsible to protect the public against health effect of global warming, almost half (41.8%) of the respondent indicated the district/municipal assemblies, because they are much closer to them. More than one-quarter (27.6%) said the central government, 18.8% said the ministry of health, and 11.9% said the World Health Organisation (WHO).

Table 9 shows the respondents' preferred methods to receive information on global warming. It is worth noting that 19.1% and 18.6% indicated that they will prefer to receive information on global warming from television and social media, respectively. However, 17.2% stated they want to obtain information about global warming from their primary care doctors, while 12.6% indicated they will rather prefer information from the World Health Organisation (WHO). Newspaper/newsletter were the least (0.5%) preferred medium to receive global warming information indicated by the respondents.

## 4. Discussion

Global warming is a serious threat to human existence. The present study sought to investigate Ghanaians' knowledge of the impact of global warming on human health. One major setback to addressing climate change issues is insufficient knowledge among people in societies [14]. Hence, knowing the gap in people's knowledge and understanding of global warming impacts on their health is essential for its adaptation and minimization.

The present study is aimed at evaluating Ghanaians' knowledge of the impact of global warming on human health. Demographic profile of the respondents revealed most of the respondents (78.8%) are educated, and agewise, most of them are relatively young (between 20 and 30 years). Generally, young people are more likely to suffer health complications of global warming because they will live with its consequences for longer periods of time [35]. Thus, creating awareness of global warming and its impacts to people in their youthful days is crucial.

From the finding in the study, it can be observed that the awareness and understanding of global warming correlates. Nearly all the respondents (91.3%) indicated that they had heard of global warming through a variety of media outlets such national radios, televisions, and social media. However, 84.4% these respondents understood the meaning of global warming. This study agrees with a similar study by Skalík [36] in Czech Republic where they found that over 80% of participants had climate change awareness.

The variations observed in the inconsistency between awareness and understanding of global warming could be the apparent nonexistence of global warming and climate change issues within the formal school curricula. It is worth noting that evidence from a study in the US by Meehan et al. [34] emphasizes the need to comprehensively incorporate climate change education in at least high school curricula to minimize or avoid the misconceptions surrounding it.

In the quest to investigate respondents' knowledge of global warming, it was deemed appropriate to assess their concern towards environmental issues in general which could have an indirect impact on their knowledge and attitudes towards global warming. Results showed more than half of the respondents (55.4%) were ‘very concerned' about local environmental issues, while 49.2% were concerned about global environmental issues. The education or literacy levels among the respondents could also be a key influencing factor for the high levels of global warming knowledge observed in the study. This is in consonant with a comprehensive review involving 47 countries which revealed that high education was positively correlated with people's perception about the seriousness of global warming [37].

Causal knowledge on global warming is also relevant, not only with respect to human health but also in global efforts to contain the global warming phenomenon. This is because misconceptions surrounding factors responsible for global warming exist which could have serious ramifications on people's health. Generally, respondents' perceived causes of global warming were natural processes, deforestation, act of the gods, burning of fossil fuel, and CO_2_ emission from vehicles and industries. Specifically, cutting down trees was mostly identified as a cause of global warming. Deforestation is a major contributor to global warming as it is known to account for 25% of the greenhouse gases responsible for global warming [38]. Moreover, deforestation aggravates the intensity of sunny days which increases the risk of human-related heat stress diseases [39, 40]. Natural processes, burning of fossil fuel, and CO_2_ emission as causes of global warming have been consistently reported in several literature [3, 6, 16]. However, the link of this worldwide menace as an act of the gods by a significant number of respondents could be regarded as misleading and a misconception. It could result in this cohort of people resorting to religious means in attempting to find solutions to global warming which will be a deviation from the required physical and scientific nonreligious measures.

Regarding effects of global warming on human health, respondents indicated it affected human health although knowledge pertaining to degree of global warming impact was relatively low. It was however worth noting that close to 84% of the respondents considered global warming to be either bad or very bad to human health.

In investigating respondents' knowledge on specific effects of global warming, close-ended questions were asked. Generally, respondents' answers to the close-ended questions revealed they are knowledgeable in this regard. With exception of mental health stress, more than 50% of the respondents agreed or strongly agreed to all variables representing effects of global warming. Perhaps, the mental health consequence of global warming does not have much publicity. Climate change effects on mental health are not reported often though it has a devastating impact on the mental and psychological well-being, directly and indirectly [41]. Particularly, climate change and global warming can cause people to suffer depression, anxiety, posttraumatic stress disorder (PTSD), drug abuse, and even committing suicide [42]. Mental health risks associated with climate change is rapidly on the rise [42], and as such, its awareness and education need to be prominent and extensive in Ghana.

Global warming affects all groups of people but a certain group of individuals are likely to suffer more than others. Finding from the study indicates that some respondents were of the notion that no groups of persons are more vulnerable than others which is not entirely accurate. For instance, poor and disadvantaged people could be more vulnerable to climate change than the well-to-do individuals [43]. Thus, this also revealed a gap in respondents' knowledge on global warming.

In addition to the inadequate knowledge on vulnerability to global warming, nearly half of the participants indicated that their local district assemblies should be doing more to protect them from global warming because they are relatively closer to them as compared to the central government, health ministry, and World Health Organisation (WHO). This is in variance to a study by Maibach et al. [16], where nearly half of the respondents were of the view that the government should be responsible for their protection. Observation from these results show that people could have different perceptions about agencies responsible for health issues or may not have much confidence in the designated bodies such as public health officials usually stationed in the health ministries. However, these public health officials are entrusted with providing information to the public concerning phenomena that endanger their health [16].

Regarding support and funding of global warming, most respondents were willing to support any project aimed at reducing the intensity of this worldwide menace. For funding, more than half of the respondents indicated the district assemblies and other agencies need more funds to help protect the public against the health effects of global warming.

## 5. Conclusion and Policy Implication

The study examined the public knowledge of respondents about global warming in Ghana. The findings of this paper are likely to be highly relevant to other countries and including those near to Ghana. The study undoubtedly revealed useful insights for policy directions in Ghana. The review of the literature and findings of the study show that the local and global environmental concerns of respondents have an impact on their knowledge of nuclear global warming. Results from the research show that majority of the Ghanaians who participated in the study have high awareness of global warming. Additionally, most of the respondents showed much concern towards the impact of global warming on their health. Based on these and other results, several useful points for policy in this regard are outlined.

First, the relevant health agencies and all other major stakeholders should join forces to increase publicity and education about global warming and its effects. It seemed the respondents hold their district assemblies in high regard, pertaining to issues concerning global warming and their health even more than the health ministry. Based on this, major stakeholders including public health officials could work closely with these metropolitan, municipal, and district assemblies to convey global warming education to the public. The mass media including social media could be used as a medium of communication, since it was the most preferred means of receiving information among the respondents.

Second, with regard to causes of global warming and its health impacts, certain misconceptions are still lingering. A significant number of respondents indicated global warming was an act by the gods. Also, most participants were either not sure or disagreed that global warming caused mental issues. Mental health problems linked to climate change are on the rise [42]. Hence, stakeholders and policymakers should strongly emphasize on mental health when making decisions and policies on global warming awareness creation.

In conclusion it is worth noting that the solution to climate change is not going to come from those who research and develop earth system models. It is also unlikely to come from government policy. Instead, the main driver will finally be a desire by the general public to avoid extreme changes to weather patterns. Also, the citizenry education on the effects and adaptation strategies of global warming will require a deepened public engagement of all stakeholders. As this will provide a very useful tool by which the development of effective public policies as well as citizenry participation can occur.

## Figures and Tables

**Table 1:** Demographic data of respondents.

Variables	Female, *N* (%)	Male, *N* (%)	Total, *N* (%)	Significance value
Age				
Under 20	82 (7.3)	138 (12.2)	220 (19.5)	*X* = 28.387
20-30	274 (24.2)	212 (18.8)	486 (43.0)	*P* ≤ 0.001
30-40	140 (12.4)	98(8.7)	238 (21.1)	
40-50	70 (6.2)	74 (6.5)	144 (12.7)	
50 and higher	24 (2.1)	18 (1.6)	42 (3.7)	
Total	590 (52.2)	540 (47.8)	1130 (100)	
Education				
Primary	103 (9.1)	156 (13.8)	259 (22.9)	*X* = 23.246
Secondary	120 (10.6)	87 (7.7)	207 (18.3)	*P* ≤ 0.001
Undergraduate	174 (15.4)	143 (12.7)	317 (28.1)	
Postgraduate	66 (5.8)	42 (3.7)	108 (9.6)	
Nonformal	127 (11.2)	112 (9.9)	239 (21.2)	
Total	590 (52.2)	540 (47.8)	1130 (100)	
Residence				
Rural	234 (20.7)	226 (20.0)	460 (40.7)	*X* = 0.561
Urban	356 (31.5)	314 (27.8)	670 (59.3)	*P* = 0.454
Total	590 (52.2)	540 (47.8)	1130 (100)	
Region				
Northern belt	110 (9.7)	82 (7.3)	192 (17.0)	*X* = 21.061
Middle belt	308 (27.3)	230 (20.4)	538 (47.6)	*P* ≤ 0.001
Coastal belt	172 (15.2)	228 (20.2)	400 (35.4)	
Total	590 (52.2)	540 (47.8)	1130 (100)	
Social status				
Upper class	264 (23.4)	162 (14.3)	426 (37.7)	*X* = 42.315
Middle class	296 (26.2)	302 (26.7)	598 (52.9)	*P* ≤ 0.001
Lower class	30 (2.7)	76 (6.7)	106 (9.4)	
Total	590 (52.2)	540 (47.8)	1130 (100)	

**Table 2:** Knowledge and understanding of global warming.

Variables	Female, *N* (%)	Male, *N* (%)	Total, *N* (%)	Significance value
Before this interview, have you heard of global warming				
Yes	528 (46.7)	504 (44.6)	1032 (91.3)	*X* = 6.358
No	60 (5.3)	36 (3.2)	96 (8.5)	*P* = 0.042
I do not know	2 (0.2)	0 (0.0)	2 (0.2)	
Total	590 (52.2)	540 (47.8)	1130 (100)	
Would you say you understood what global warming means				
Yes	524 (46.4)	430 (38.1)	954 (84.4)	*X* = 18.497
No	50 (4.4)	88 (7.8)	138 (12.2)	*P* ≤ 0.001
I do not know	16 (1.4)	22 (1.9)	38 (3.4)	
Total	590 (52.2)	540 (47.8)	1130 (100)	

**Table 3:** Concern about environmental issues.

Variable	Number	Percentage
Global environmental issue		
Very concerned	556	49.2
Concerned	488	43.2
Not concerned	72	6.4
Not at all concerned	14	1.2
Local environmental issues		
Very concerned	626	55.4
Concerned	446	39.5
Not concerned	44	3.9
Not at all concerned	14	1.2

**Table 4:** Effect of global warming in human health.

Variable	Female, *N* (%)	Male, *N* (%)	Total, *N* (%)	Significance value
Does global warming have an impact on human health?				
Yes	528 (46.7)	414 (36.6%)	942 (83.4)	*X* = 42.558
No	42 (3.7)	54 (4.8)	96 (8.5)	*P* ≤ 0.001
I do not know	20 (1.8)	72 (6.4)	92 (8.1)	
Total	590 (52.2)	540 (47.8)	1130 (100)	
Do you think global warming is bad or good to human health?				
Very bad	282 (25.0)	274 (24.2)	556 (49.2)	*X* = 63.879
Bad	232 (20.5)	160 (14.2)	392 (34.7)	*P* ≤ 0.001
Good	30 (2.7)	2 (0.2)	32 (2.8)	
Very good	2 (0.2)	22 (1.9)	24 (2.1)	
I do not know	44 (3.9)	82 (7.3)	126 (11.2)	
Total	590 (52.2)	540 (47.8)	1130 (100)	

**Table 5:** Do you think the following have been caused by global warming.

Variable	Strongly agree, *N* (%)	Agree, *N* (%)	Neither agree/disagree, *N* (%)	Disagree, *N* (%)	Strongly disagree, *N* (%)
Heat stroke from extreme hot temperatures	568 (50.3)	342 (30.3)	60 (5.3)	144 (12.7)	16 (1.4)
Malnutrition/food reduction/hunger	364 (32.2)	452 (40.0)	174 (15.4)	88 (7.8)	52 (4.6)
Vectorborne illness such as malaria and cholera	214 (18.9)	436 (38.6)	222 (19.6)	168 (14.9)	90 (8.0)
Heart disease	164 (14.5)	426 (37.7)	196 (17.3)	214 (18.9)	130 (11.5)
Contaminated water	458 (40.5)	320 (28.30)	180 (15.9)	138 (12.2)	34 (3.0)
Drought/water shortage/fires	578 (51.2)	342 (30.3)	116 (10.3)	68 (6.0)	26 (2.3)
Lung disease/asthma/respiratory problems	328 (29.0)	292 (25.8)	140 (12.4)	100 (8.8)	270 (23.9)
Flooding and downpours	602 (53.3)	258 (22.8)	120 (10.6)	136 (12.0)	14 (1.2)
Mental health, stress	158 (14.0)	180 (15.9)	174 (15.4)	262 (23.2)	356 (31.5)
Pollution/air pollution/air quality	738 (65.3)	204 (18.1)	70 (6.2)	52 (4.6)	66 (5.8)

**Table 6:** Groups vulnerable to health impact of global warming.

People who are vulnerable	Number (*N*)	Percentage (%)
Infant/young children	906	11.7
City or urban dwellers	846	11.0
The poor	852	11.0
Outdoor workers	886	11.5
People living with light or sensitive skin	944	12.2
Residents in the coast or flood-prone areas	816	10.6
The sick, disabled, obese and low immunity	932	12.1
Everyone	606	12.1
No group are more vulnerable than others	506	6.6
Not sure	426	5.5

**Table 7:** If nothing is done in the next 5-10 years, which of the following will become more or less common in your community.

Variable	Much more common, *N* (%)	Somewhat/a little more common, *N* (%)	About the same, *N* (%)	A little less common, *N* (%)
Air pollution	1036 (91.7)	76 (6.7)	2 (0.2)	16 (1.4)
Asthma and other lungs	640 (56.6)	432 (38.2)	16 (1.4)	42 (3.7)
Heat stroke	806 (71.3)	196 (17.3)	86 (7.6)	42 (3.7)
Hunger/ malnutrition	640 (56.6)	178 (15.8)	254 (22.5)	58 (5.1)
Disease carried by insects	656 (58.1)	150 (13.3)	282 (25.0)	42 (3.7)
Illness from food/waterborne disease	672 (59.5)	138 (12.2)	260 (23.0)	60 (5.3)
Death	650 (57.5)	148 (13.1)	288 (25.5)	42 (3.7)
Cancer	570 (50.4)	186 (16.5)	302 (26.7)	72 (6.4)

**Table 8:** Respondents' support for funding and willingness to support efforts to reduce global warming intensity.

Variable	Number (*N*)	Percentage (%)
Are you willing to support to reduce global warming intensity?		
Yes	888	78.6
No	32	2.8
May be	154	13.6
I do not think so	56	5.0
Total	1130	100.0
Do you think increase funding to the region and district and other health agencies would help to protect against the health effect of global warming?		
Yes	664	58.8
No	106	9.4
May be	212	18.8
I do not think so	148	13.1
Total	1130	100

**Table 9:** Respondents' preferred methods to receive information on global warming.

Variables	Number (*N*)	Percentage (%)
Primary care doctors	194	17.2
Family and friends	30	2.7
World health	142	12.6
Television	216	19.1
Religious leaders	144	12.7
Social media	210	18.6
NGO	12	1.1
Newsletters	6	0.5
Billboards/posters	46	4.1
NCCE	40	3.5
Environmental Protection Agency	62	5.5
National radio	28	2.5
Total	1130	100

## Data Availability

The data used to support the finding of this study are included within the article.
